# Rapid specific detection of oral bacteria using Cas13-based SHERLOCK

**DOI:** 10.1080/20002297.2023.2207336

**Published:** 2023-05-11

**Authors:** Jett Liu, Camden Carmichael, Hatice Hasturk, Wenyuan Shi, Batbileg Bor

**Affiliations:** a Department of Microbiology, The Forsyth Institute, Cambridge, MA, USA; b Center for Clinical and Translational Research, The Forsyth Institute, Cambridge, MA, USA; c Department of Oral Medicine, Infection and Immunity, Harvard School of Dental Medicine, Boston, MA, USA

**Keywords:** diagnostics, Sherlock, rapid detection, oral bacteria, CRISPR-cas

## Abstract

Decades of ongoing research has established that oral microbial communities play a role in oral diseases such as periodontitis and caries. Yet the detection of oral bacteria and the profiling of oral polymicrobial communities currently rely on methods that are costly, slow, and technically complex, such as qPCR or next-generation sequencing. For the widescale screening of oral microorganisms suitable for point-of-care settings, there exists the need for a low-cost, rapid detection technique. Here, we tailored the novel CRISPR-Cas-based assay SHERLOCK for the species-specific detection of oral bacteria. We developed a computational pipeline capable of generating constructs suitable for SHERLOCK and experimentally validated the detection of seven oral bacteria. We achieved detection within the single-molecule range that remained specific in the presence of off-target DNA found within saliva. Further, we adapted the assay for detecting target sequences directly from unprocessed saliva samples. The results of our detection, when tested on 30 healthy human saliva samples, fully aligned with 16S rRNA sequencing. Looking forward, this method of detecting oral bacteria is highly scalable and can be easily optimized for implementation at point-of-care settings.

## Introduction

Oral polymicrobial dysbiosis has been well established as an etiology for numerous oral diseases, while a healthy oral microbiome has been suggested as crucial to preventing oral and systemic diseases [1–7]. In the case of periodontitis, a specific subgroup of microorganisms termed the red complex has been strongly associated with disease [1]. For caries, multiple acid-producing bacteria have been implicated in cavity formation [8,9]. Furthermore, specific bacteria such as *Streptococcus mutans*, *Porphyromonas gingivalis*, and *Fusobacterium nucleatum* are more intensely studied and have been highly associated with oral diseases [7,10–12]. These bacteria and others are also implicated in systemic diseases including various cancers, digestive diseases, cardiovascular diseases [3], and neurodegenerative diseases [13,14].

To detect microbes residing in the oral cavity, current methods frequently rely on quantitative polymerase chain reaction (qPCR) or next-generation sequencing (NGS). These techniques often require shipment to a central laboratory, high processing costs, and specialty technicians to interpret the data. Due to these limitations, despite the ubiquitous use of these techniques among the scientific community, large-scale and widespread employment of qPCR or sequencing-based methods for profiling oral microbes has remained limited [15,16]. To facilitate precision oral medicine that can effectively treat conditions associated with oral bacteria, there is a clear need for the field of oral health to move towards novel diagnostics suitable for widespread use. To this end, we propose a recently developed detection method, Specific High-sensitivity Enzymatic Reporter Unlocking (SHERLOCK) [17,18], as a means to detect oral bacteria in a rapid, highly modular fashion.

SHERLOCK is a nucleic acid detection technique that leverages the CRISPR-Cas Class II enzyme Cas13a. It has been recently employed to detect a variety of pathogens including SAR-CoV-2 [19], various strains of ZIKA and Dengue virus [18], the malaria-causing parasites *P. falciparum* and *P. vivax* [20], and bacterial pathogens such as *Escherichia coli and Pseudomonas aeruginosa* [18]. SHERLOCK detection of nucleic acids consists of four key steps: (1) amplification of a target region; (2) Cas13a CRISPR-RNA (crRNA) recognition of the target region; (3) cleavage of reporter RNAs by Cas13a; and (4) fluorescence readout of cleaved reporter RNAs. The first step, amplification of a target region, utilizes the isothermal amplification technique Recombinase Polymerase Amplification (RPA) [21,22] to generate many copies of a target amplicon. Next, a crRNA, bound to Cas13a in a complex, recognizes a 28 base pair (bp) region on the target amplicon, a protospacer [18]. Upon crRNA binding to the protospacer, Cas13a exhibits indiscriminate RNase activity, cleaving nearby RNAs [18]. SHERLOCK leverages the collateral cleavage activity of Cas13a by including reporter RNAs within the reaction that fluoresce when cleaved [17]. This fluorescent signal can be quantified over time and equated to detection of the specific target sequence.

In combining Cas13a detection with RPA, SHERLOCK is capable of rapid detection in the single-molecule range without the need for complex equipment [12]. Furthermore, SHERLOCK reagents, including crRNAs and primers, can be lyophilized and employed on a laminar flow strip for an estimated cost of $0.60 per single-plex assay [17,18]. These improvements do not compromise the sensitivity of the assay compared to current gold-standard nucleic acid detection methods such as qPCR and ddPCR (droplet digital PCR) [18]. For these reasons, SHERLOCK and similar CRISPR-Cas-based assays have been proposed as ideal detection tools for future diagnostics [23].

To our knowledge, SHERLOCK has not yet been applied to the detection of oral bacteria. Here, we present the adaptation of SHERLOCK to detect oral bacteria by designing RPA primer pairs and crRNAs that target conserved, species-specific genes encoded within target bacteria. We experimentally validate detection using our designed constructs for seven oral bacteria and display that our crRNAs and primer pairs are highly specific and sensitive. We further tailor the SHERLOCK methodology to detect bacteria within unprocessed saliva samples. These results serve as a proof of principle that SHERLOCK can be a facile, rapid, and highly precise detection method for oral microorganisms with the potential to be further scaled and developed for use in point-of-care settings.

## Materials and methods

### Computational pipeline for species-specific primer and crRNA design

Given a bacterial species, our pipeline extracts all MetaPhlAn 4.0 marker genes attributed to that particular species [24]. The pipeline divides each marker gene into every possible 28 bp protospacer. For each possible protospacer, the pipeline searches for ancillary primers compatible with RPA using Primer3 [25] with the previously described thresholds of [17] primer length between 25 and 35 bp; Tm between 54 and 67°C; and GC% between 20 and 80%. To the 5’ end of each left primer, a T7 polymerase promoter sequence is added (5’-AATTCTAATACGACTCACTATAGGGTCCA-3’) [17]. crRNA sequences are then generated from each protospacer by adding a crRNA backbone sequence (5’-GGGGAUUUAGACUACCCCAAAAACGAAGGGGACUAAAAC-3’) [18] to the 5’ of the reverse complement of the protospacer.

For each candidate crRNA and its corresponding primer pair (hereafter referred to as a crRNA-primer set), the putative RNA amplicon produced by RPA and T7 transcription is predicted. The minimum free energy (MFE) of the RNA amplicon secondary structure is computed using ViennaRNA v2.5.1 [26]. As a higher secondary structure MFE of the crRNA target has been suggested to increase crRNA-protospacer binding [17,18], crRNA-primer sets are sorted in decreasing RPA amplicon secondary structure MFE. For the synthesis of crRNAs, a ssDNA template sequence is generated by adding a T7 promoter sequence to the 3’ end of the reverse complement of the crRNA sequence (5’-TATAGTGAGTCGTATTAATTTC-3’).

Using our computational pipeline, we synthesized seven crRNA-primer sets, each targeting one of seven oral bacteria (Table 1). All primers and crRNA-ssDNA templates were ordered from Integrated DNA Technologies (IDT). crRNAs were synthesized using a ssDNA template, a T7 primer (5’ - GAAATTAATACGACTCACTATAGGG − 3’), and the HiScribe T7 kit (NEB, #E2050S).

**Table 1.** Bacteria targeted in this study and their disease association, genome size, and growth conditions.

Bacterial Species	Strain Name	Disease Association	Genome Size (bp)	Growth Conditions	References
*Streptococcus mutans* (Sm)	F0577	Caries	2030936	Brain Heart Infusion Medium 23% O_2_, 77% N_2_	[27,28]
*Scardovia wiggsiae* (Sw)	F0424	Caries	1550000	Brain Heart Infusion Medium 0% O_2_, 5% CO_2_, 95% N_2_	[8,29]
*Aggregatibacter actinomycetemcomitans* (Aa)	AA075	Periodontitis	2260000	Brain Heart Infusion Medium 23% O_2_, 77% N_2_	[30,31]
*Porphyromonas gingivalis* (Pg)	ATCC33277	Periodontitis	2378872	Brain Heart Infusion Medium 0% O_2_, 5% CO_2_, 95% N_2_	[32,33]
*Acinetobacter baumannii* (Ab)	19606	Oral and Systemic Disease	3824000	Brain Heart Infusion Medium 23% O_2_, 77% N_2_	[34,35]
*Klebsiella pneumoniae* (Kp)	Fpn6806	Oral and Systemic Disease	5491870	Brain Heart Infusion Medium 0% O_2_, 5% CO_2_, 95% N_2_	[36–38]
*Staphylococcus aureus* (Sa)	F0253A	Oral and Systemic Disease	2874302	Brain Heart Infusion Medium 23% O_2_, 77% N_2_	[34,39,40]

### Growth of target bacteria and gDNA isolation

Seven target bacteria were grown in the conditions listed in Table 1. Genomic DNA (gDNA) of each target bacterium was isolated using a previously described method [41]. Briefly, the MasterPure gram-positive DNA purification kit (Biosearch, #MC85200) was used according to the manufacturer’s protocol with the addition of a bead beating step (3 × 30 s at 6 m/s with 1-min pause intervals). Isolated gDNA concentrations were measured using the Qubit Broad Range dsDNA kit (Invitrogen, #Q32850). In the case of isolated gDNA from axenic cultures, gDNA concentration and copy number/uL were calculated based on established genome lengths (Table 1).

### One-pot SHERLOCK

SHERLOCK detection was performed in a one-pot reaction, according to a previously described protocol [17], in which the *Leptotrichia wadei* ortholog of Cas13a (*lw*Cas13a), RPA reagents, primers, crRNA, and several adjuvant reagents are combined. Reagents were combined in the specific order and at volumes listed in Appx. Table A1 then pulse mixed for 3 s. RPA reagents from the TwistAmp Basic Kit (TwistDx, #TABAS03KIT) were used. A Spectra Max iD3 Multi-Mode microplate reader was preheated to 37°C. Target gDNA was diluted to the desired concentrations and was added at a volume of 4 µL into 20.6 µL of the Cas-RPA mix. The final reaction mixture was homogenized and centrifuged at 500 *g* for 30 s before 20 µL was pipetted into a 384-well optical plate (Roche, #5102430001). The plate was loaded into the microplate reader and fluorescence readings of each well were taken at 485/528 nm using kinetic reads at 5-min intervals.

### Bacterial detection directly from unprocessed saliva samples

To adapt the SHERLOCK system for the detection of bacteria in unprocessed saliva samples (without a DNA extraction step), we modified a previously described SHERLOCK protocol [19] in which EGTA (ethylene glycol-bis(beta-aminoethyl ether)-*N,N,N*’,*N*’-tetraacetic acid) and DTT (Dithiothreitol) are added to a saliva sample. Briefly, ETGA and DTT were added at a concentration of 500 mM and 1 mM, respectively, to an unprocessed saliva sample. The saliva mixture was then heated at 95°C for 15 min. The saliva mixture, in lieu of target gDNA, was then used in a one-pot SHERLOCK assay (this methodology is hereafter referred to as SHERLOCK-EGTA+DTT).

In our spiked saliva experiments, to determine the amount of spiked target bacteria, we estimated the CFU/µL of live bacteria using previously established optical density to CFU measurements for both Pg [42] and Sm [43]. Unprocessed saliva samples were collected from medically healthy subjects with no oral diseases or conditions (see below).

### Clinical sample collection and validation

We collected unstimulated saliva samples (1.5 mL) from 30 adults (18 females, 12 males) with documented medical, dental, and periodontal health. As previously described [44], participants were excluded if they received periodontal disease treatment within 3 months, antibiotic treatment within 8 weeks, or antimicrobial mouthwash treatment within 4 weeks. Additionally, participants with less than 10 teeth or those currently using dentures were excluded. The study received Forsyth Institutional Review Board approval (#18–06, Dr Hasturk), and all subjects provided written informed consent. Saliva samples were split into two aliquots: one for 16S rRNA sequencing and one for SHERLOCK-EGTA+DTT detection. gDNA was isolated from an aliquot of each saliva sample, and V3-V4 16S rRNA sequencing was performed on all 30 samples (Zymogen). 16S rRNA taxonomic assignments and relative abundances measurements were determined by comparing in-house Zymogen analyses with 16S rRNA sequences from the expanded Human Oral Microbiome Database (eHOMD) [45]. Briefly, the in-house Zymogen analysis utilizes DADA2 [46] to determine amplicon sequence variants (ASVs) and ASV relative abundances within each sample. The Zymogen generated ASVs were then compared to the eHOMD 16S rRNA database for taxonomic assignment as previously described [41]. To generate *Streptococcus* and *Porphyromonas* phylogenetic trees, *Streptococcus* and *Porphyromonas* 16S rRNA sequences were downloaded from eHOMD. One 16S rRNA sequence was selected for each Human Microbial Taxon (HMT), and the 16S rRNA sequence of *Escherichia coli* from eHOMD was included as an outgroup. Sequences were aligned using MAFFT [47] and trimmed using TrimAl [48]. Maximum-likelihood trees were inferred using IQ-tree v2.1.4-beta with ultrafast bootstrap (−bb 1500) [49]. For SHERLOCK-EGTA+DTT detection, the second aliquot of each saliva sample was diluted 1:4 with sterile PBS before addition to SHERLOCK reagents.

## Results

### Computational pipeline generates constructs compatible with SHERLOCK

**Figure 1.**
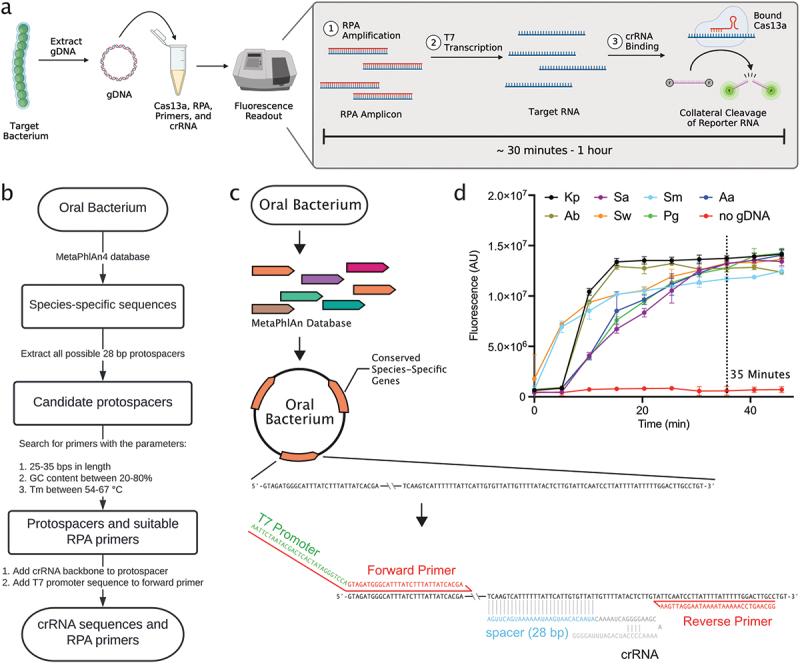
Computational pipeline to generate crRnas and primer pairs targeting conserved species-specific genes. a. Visual schematic of one-pot SHERLOCK (Appx Table 1). b. Workflow of the computational pipeline that generates crRnas and primers for specific bacterial detection using SHERLOCK. c. Schematic illustrating the architecture of crRNA and primer design for SHERLOCK. d. Detection of target gDNA using each synthesized crRNA. Target gDNA was added at a concentration of 1 picomolar (pM) per reaction, equating to 30,000 targets per µL reaction. Each crRNA targeted one of seven oral bacterial targets: *Streptococcus mutans* (Sm), *Klebsiella pneumoniae* (Kp), *Aggregatibacter actinomycetemcomitans* (Aa), *Acinetobacter baumannii* (Ab), *Staphylococcus aureus* (Sa), *Scardovia wiggsiae* (Sw), or *Porphyromonas gingivalis* (Pg).

To detect a specific nucleic acid sequence, SHERLOCK (Figure 1a, Appx. Table A1) requires a set of three constructs specific to the target sequence (hereafter referred to as a crRNA-primer set): a crRNA, a forward primer for RPA, and a reverse primer for RPA. To design crRNA-primer sets for the SHERLOCK detection of oral bacteria, we compiled a computational pipeline (Figure 1b) that generates crRNA-primer sets targeting conserved species-specific genes (Figure 1c, *Materials and Methods*).

To experimentally validate our computational pipeline, we synthesized seven crRNA-primer sets, each targeting a species-specific gene encoded within one of seven oral bacteria. We targeted *Streptococcus mutans* (Sm), *Porphyromonas gingivalis* (Pg), *Scardovia wiggsiae* (Sw), *Aggregatibacter actinomycetemcomitans* (Aa), *Klebsiella pneumoniae* (Kp), *Acinetobacter baumannii* (Ab), and *Staphylococcus aureus* (Sa) for detection (Table 1, Appx. Table A2). As an assessment of the seven crRNA-primer sets, we tested each crRNA-primer set with SHERLOCK in the presence of genomic DNA (gDNA) isolated from their intended bacterial target. All seven crRNA-primer sets produced a target-specific fluorescent signal over time. At 35 min of detection, the typical time at which the fluorescent signals plateaued, the fluorescent signals were greater than 19-fold change in the presence of target gDNA (1 picomolar (pM); 30000 copies/µL reaction) as compared to a negative control containing no target gDNA (Figure 1d). These results confirmed that our computational pipeline produced crRNA-primer sets compatible with SHERLOCK detection.

### Designed crRNAs display specific and sensitive signal

**Figure 2.**
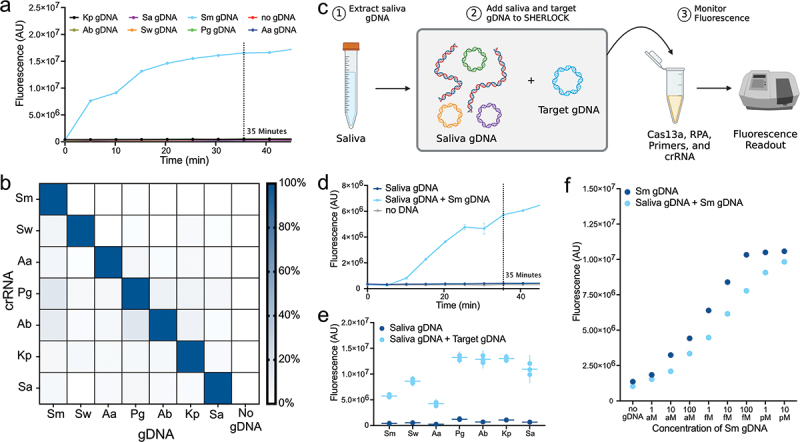
Specificity and sensitivity of designed crRNA and primer pairs. a. Representative fluorescent trace plot displays the specificity of the Sm crRNA-primer set against other bacterial targets. b. Heat map displaying cross-reactivity of synthesized crRNA-primer sets against all tested oral bacterial targets. The min-max linear normalized signal at 35 min of detection is displayed. Target gDNA was added at a concentration of 1 pM per reaction. All other corresponding individual plots can be found in Appx. Fig. 1. c. Visual schematic displaying specificity assays with saliva gDNA. d. Representative example fluorescence trace plot displays the specificity of the Sm crRNA-primer set against background saliva gDNA. Other individual plots can be found in Appx. Fig. 2. e. the specificity of synthesized crRNA-primer sets against saliva gDNA. Signal at 35 min of detection is displayed. Each point represents a technical replicate. 10 ng of background saliva gDNA was added per reaction. Target gDNA was added at a concentration of 1 pM per reaction. f. SHERLOCK detection at 35 min of decreasing 10-fold concentrations of Sm gDNA alone or combined with 10 ng of saliva gDNA (Appx. Fig. 3).

With our seven synthesized crRNA-primer sets displaying signal in the presence of their target sequence, we next sought to examine the specificity and sensitivity of our designed crRNA-primer sets. As a preliminary measure of the specificity of the designed crRNA-primer sets, we tested the cross-specificity of each crRNA-primer set against isolated gDNA (1 pM) from each of the seven bacterial targets (Figure 2a). At 35 min of detection, when in the presence of gDNA isolated from their intended target, all crRNA-primer sets produced a fluorescent signal greater than 10-fold change higher than off-target reactions (Figure 2b, Appx. Figure A1). These cross-specificity assays indicated that when tested against gDNA from several off-target oral bacteria, all designed crRNA-primer sets produced a clear and discriminant signal only against their intended target.

Next, to account for the hundreds of endogenous species within saliva, we conducted a more thorough interrogation of the specificity of each crRNA-primer set by testing them against a background of saliva gDNA (Figure 2c). We surveyed the fluorescence signal at 35 min of detection produced by each crRNA-primer set when tested against (1) saliva gDNA (10 ng); (2) saliva gDNA (10 ng) spiked with target bacterial gDNA (1 pM); and (3) no saliva or target bacterial gDNA (Figure 2d). Each crRNA-primer set displayed a greater than 12-fold change increase in fluorescent signal when in the presence of gDNA from their bacterial target as compared to saliva gDNA alone (Figure 2e; Appx. Figure A2). Intriguingly, at 35 min of detection, the different crRNA-primer sets displayed differing levels of fluorescent signal, indicating that they may express varying levels of efficiency (Figure 2e). Altogether, these results validated the specificity of our crRNA-primer sets in the presence of off-target oral bacterial and human gDNA.

**Figure 3.**
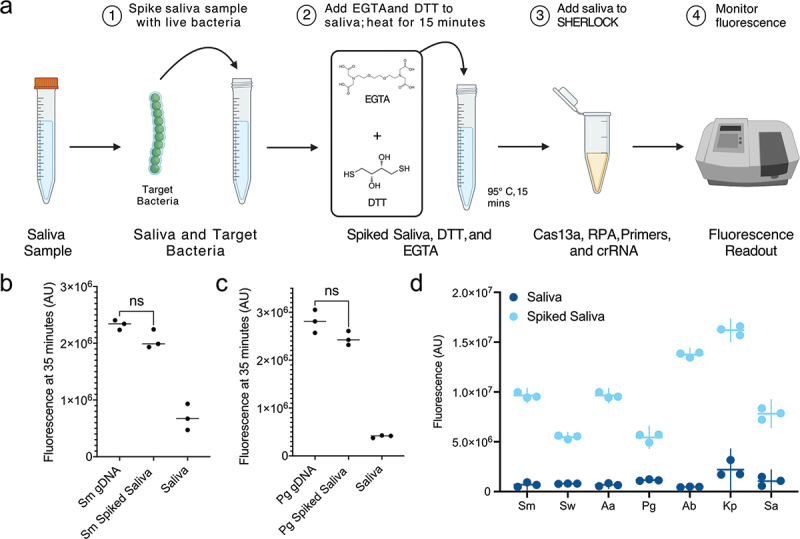
Oral pathogen detection directly from saliva samples. a. Visual schematic of unprocessed saliva detection with SHERLOCK-EGTA+DTT. b-c. Fluorescent signal at 35 min produced by SHERLOCK with Sm (B) or Pg (C) gDNA and SHERLOCK-EGTA+DTT spiked with an equivalent concentration of live Sm or Pg cells (Appx. Fig. 4). d. Specificity and fluorescent signal of crRNA-primer sets when used with SHERLOCK-EGTA+DTT on unprocessed saliva samples. Saliva was spiked at a concentration of 300 target bacterial cells per µL reaction. Fluorescent signal at 35 min is displayed. Each point represents a technical replicate. All plots can be found in Appx. Fig. 5.

The addition of off-target saliva gDNA may also affect the sensitivity of SHERLOCK detection. To assess this, we performed SHERLOCK detection against a concentration gradient of Sm gDNA ranging from 1 attomolar (aM) (0.5 copies/µL reaction) to 10 pM (300,000 copies/µL reaction) in two conditions: (1) with 10 ng of background saliva gDNA and (2) without any background gDNA. Across the Sm gDNA dilutions, the saliva gDNA background dampened the fluorescent signal strength by an average of 20% at 35 min of detection (Figure 2f, Appx. Figure A3). Despite this decrease in signal strength incurred by the addition of saliva gDNA, at our lowest concentration of Sm gDNA, 1 aM (0.5 copies/µL reaction), we still achieved a fluorescent signal 1.4-fold change greater with Sm gDNA than without Sm gDNA in a background of saliva gDNA (Appx. Figure 3). The ability of our assay, therefore, to detect target sequences down to a concentration of 1 aM was not hindered by the addition of a saliva gDNA background. Further, in assessing the detection signal at 35 min across dilutions of target gDNA, there is a clear correlation between target DNA concentration and measured signal (Figure 2f).

### Detection of bacteria from unprocessed saliva

With our synthesized crRNA-primer sets displaying high specificity and sensitivity, we modified a previously described protocol for SHERLOCK on unprocessed saliva samples [19] to extend the utility of our bacterial detection assay (Figure 3a, *Materials and Methods*). EGTA and DTT were added to inhibit the saliva RNases while a 95°C treatment was added to lyse bacteria. We hereafter refer to this enhanced method as SHERLOCK-EGTA+DTT.

We sought to assess how the SHERLOCK-EGTA+DTT detection of live bacteria in unprocessed saliva samples compared to SHERLOCK detection of isolated gDNA. We chose to compare the two methodologies using gram-negative Pg and gram-positive Sm. At 35 min of Sm or Pg detection, when equivalent amounts of cfu/µL and gDNA copy numbers (300 cfu/µL reaction; 300 copies/µL reaction) were added to SHERLOCK-ETGA+DTT and SHERLOCK, respectively, we did not observe a significant difference between the fluorescent signals produced by the two methodologies (Figure 3b,c, Appx. Figure A4). Therefore, in the case of Sm and Pg detection, the SHERLOCK-EGTA+DTT methodology is as effective as SHERLOCK is at detecting target nucleic acid sequences from isolated gDNA.

We tested the ability of each of the seven synthesized cRNA-primer sets using SHERLOCK-EGTA+DTT to detect their target bacterium from unprocessed saliva. All seven crRNA-primer sets with SHERLOCK-EGTA+DTT produced a fluorescent signal greater than 6-fold change higher when tested against unprocessed saliva spiked with live target bacterium (300 cfu/µL reaction) compared to unprocessed saliva without the target bacteria (Figure 3d, Appx. Figure A5). As observed in our isolated gDNA detection (Figure 2e), different crRNA-primer sets displayed varying levels of fluorescent signal at 35 min of detection (Figure 3d). However, this did not affect detection results. In no reaction did unprocessed saliva samples without spiked target bacteria produce a fluorescent signal greater than 19% of samples with the target bacteria (Appx. Figure A5). These two observations indicated that our added reagents and additional heating step in SHERLOCK-EGTA+DTT effectively neutralized endogenous RNase activity and lysed bacterial cells. Additionally, these data indicate that our designed crRNA-primers sets remained effective when used for detection from unprocessed saliva samples.

### Detection of target bacteria in unprocessed human saliva aligns with 16S rRNA sequence profiling

**Figure 4.**
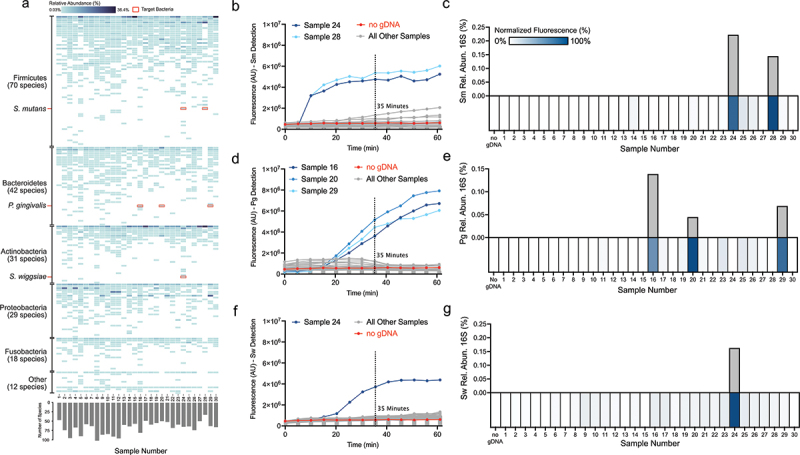
16S rRNA sequencing validation of SHERLOCK-EGTA+DTT detection on healthy subject saliva. a. 16S rRNA taxonomic predictions for 30 healthy saliva samples. b. SHERLOCK-EGTA+DTT detection of 30 healthy subject samples using the Sm crRNA-primer set. Six microlitres of 1:4 diluted healthy subject saliva was added per reaction. c. 16S rRNA sequencing compared to SHERLOCK-EGTA+DTT Sm detection. Upper bar graph displays 16S rRNA relative abundance (%) of Sm. Lower heatmap displays the min-max linear normalized signal of Sm SHERLOCK-EGTA+DTT detection at 35 min. d. SHERLOCK-EGTA+DTT detection of 30 healthy subject samples using the Pg crRNA-primer set. Six microlitres of 1:4 diluted healthy subject saliva were added per reaction. e. 16S rRNA sequencing compared to SHERLOCK-EGTA+DTT Pg detection. Upper bar graph displays 16S rRNA relative abundance (%) of Pg. Bottom heatmap displays the min-max linear normalized signal of Pg SHERLOCK-EGTA+DTT detection at 35 min. f. SHERLOCK-EGTA+DTT detection of 30 healthy subject samples using the Sw crRNA-primer set. Six microlitres of 1:4 diluted healthy subject saliva were added per reaction. g. 16S rRNA sequencing compared to SHERLOCK-EGTA+DTT Sw detection. Upper bar graph displays 16S rRNA relative abundance (%) of Sw. Bottom heatmap displays the min-max linear normalized signal of Sw SHERLOCK-EGTA+DTT detection at 35 min.

To be effective in healthcare and research settings, it is important that a novel detection method is similar in sensitivity and accuracy to established methods such as 16S rRNA sequencing [15,16,50]. To demonstrate that SHERLOCK-EGTA+DTT detection is similar in detection sensitivity to 16S rRNA sequencing, we collected 30 saliva samples from healthy subjects and performed 16S rRNA sequencing on each sample. Within the 30 saliva samples, 16S rRNA sequencing identified 202 different oral bacterial species (Figure 4a). These saliva samples each contained between 33 and 102 oral bacterial species, a typical range found in other studies [51]. Of our seven candidate oral bacteria, 16S rRNA sequencing identified two (samples 24 and 28), three (samples 16, 20, and 29), and one (sample 24) samples positive for Sm, Pg, and Sw, respectively (Figure 4a). None of the other four bacterial targets (Aa, Kp, Sa, and Ab) were identified in any of the 30 samples by 16S rRNA sequencing. This was not surprising given that these bacteria are rarely found in healthy oral cavities [52].

We performed SHERLOCK-ETGA+DTT on all 30 samples using the Sm, Pg, and Sw crRNA-primer sets. By Sm detection, samples 24 and 28 produced a signal greater than 8-fold change over a no gDNA control, while no other samples produced a signal greater than 1.8-fold change over the negative control (Figure 4b). These results completely aligned with the 16S rRNA detection of Sm (Figure 4c). Similarly, by Pg detection, we observed only three strong fluorescent signals (all greater than sixfold change over the negative control) (Figure 4d). The three signals were from the same samples that were identified to contain Pg by 16S rRNA sequencing (Figure 4e). With Pg crRNA, we also observed the lowest relative abundance by 16S rRNA sequencing, 0.05%, was discernible by SHERLOCK-EGTA+DTT, illustrating the high sensitivity of the assay. By Sw detection, we observed only one discernible signal: the signal produced by sample 24 was 6.5-fold change greater than the negative control (Figure 4f). This was the same sample identified to contain Sw by 16S rRNA sequencing (Figure 4g). Altogether, our SHERLOCK-ETGA+DTT detection results accurately recapitulated the 16S rRNA sequencing results for Sm, Pg, and Sw presence (100% agreement between the two methods).

**Figure 5.**
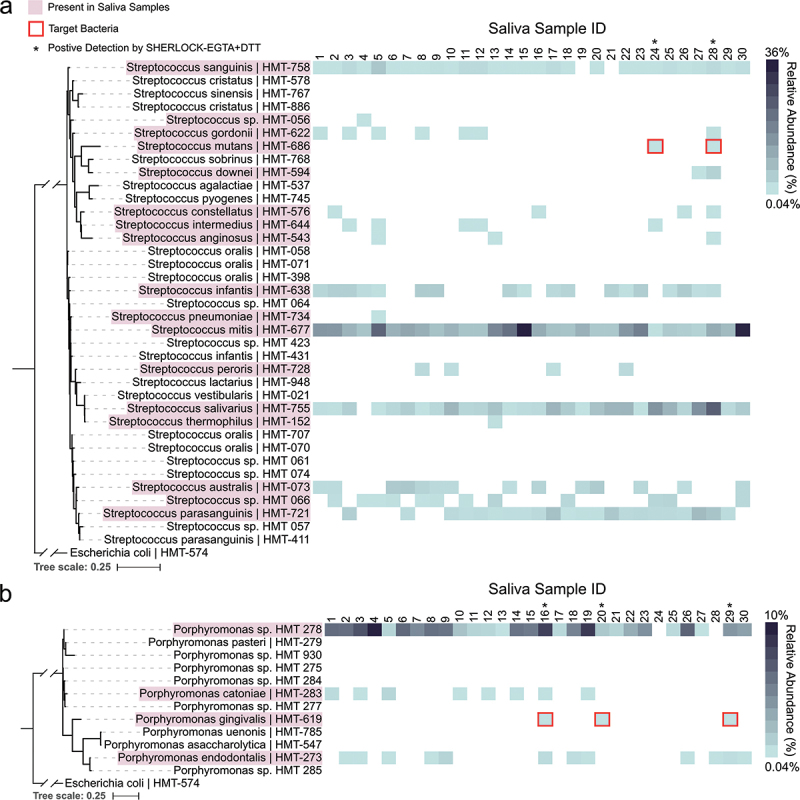
Species specificity of *S. mutans* and *P. gingivalis* detection. Pink outlines denote HMTs that were detected within our 30 saliva samples. Blue heatmaps display the relative abundance of the detected HMTs within each of the 30 saliva samples. a. Maximum-likelihood 16S rRNA phylogenetic tree of eHOMD *Streptococcus* HMTs. Asterisk denotes samples that displayed fluorescent signal in Fig. 4b,c when tested with SHERLOCK-EGTA+DTT and the designed Sm crRNA. *E. coli* is included as an outgroup. Branches leading to *E. coli* and the *Streptococcus* genus are truncated to improve readability. b. Maximum-likelihood 16S rRNA phylogenetic tree of eHOMD *Porphyromonas* HMTs. Asterisk denotes samples that displayed fluorescent signal in Fig. 4d,e when tested with SHERLOCK-EGTA+DTT and the designed Pg crRNA. *E. coli* is included as an outgroup. Branches leading to *E. coli* and the *Porphyromonas* genus are truncated to improve readability.

To further assess the species-specificity of our designed crRNAs, we surveyed the community composition of the 30 saliva samples and detected multiple species of *Streptococcus* and *Porphyromonas* (Figure 5). Of the 37 *Streptococcus* oral Human Microbial Taxons (HMTs – defined as 98.5% sequence identity between 16S rRNA genes) within the expanded Human Oral Microbiome Database (eHOMD) [45], we detected 17 *Streptococcus* HMTs within our 30 saliva samples (Figure 5a). The 17 detected *Streptococcus* HMTs are not confined to a specific clade and capture a large range of the oral *Streptococcus* genus. As evidence of the species specificity of our designed crRNAs, only samples containing *S. mutans* (samples 24 and 28) displayed a fluorescent signal when tested using SHERLOCK-EGTA+DTT with our Sm crRNA despite the presence of many *Streptococcus* HMTs across the thirty saliva samples (Figure 4b and c). Samples containing closely related *Streptococcus* species such as *S. downei* but not *S. mutans* (sample 27) did not exhibit detection by our Sm crRNA. As additional evidence, our designed Pg crRNA displayed similar specificity. Of the 12 eHOMD *Porphyromonas* HMTs, we detected four within our saliva samples (Figure 5b). Only the samples containing *P. gingivalis* (samples 16, 20, and 29) exhibited a fluorescent signal when tested with SHERLOCK-EGTA+DTT and our Pg crRNA (Figure 4d and e). Although we did not test the species-specificity of the other five crRNAs, these two examples demonstrate that our designed crRNAs, in the case of Sm and Pg detection and with respect to the species we identified within our saliva samples, are indeed species-specific.

## Discussion

While SHERLOCK has been used previously to detect pathogens and parasites, this study marks the first time it has been applied to detect oral bacteria. We designed a computational pipeline capable of generating species-specific crRNAs and primer pairs for SHERLOCK and demonstrated highly specific and sensitive detection for seven oral bacteria. Further, we tailored SHERLOCK for the detection of oral bacteria directly from unprocessed saliva samples and validated this enhanced method using healthy oral saliva samples. Our results suggest that crRNA-primer sets paired with SHERLOCK-EGTA+DTT are on par with canonical oral microbial detection techniques such as 16S rRNA sequencing. Alternative oral bacterial detection technologies and services that come closest to point-of-care settings are, to our knowledge, OralDisk [53], OralDNA Labs [54], and Bristle [16]. However, these approaches are based on either qPCR or NGS techniques. They therefore require complicated hardware, significant amounts of reagent, and up to 7 days of processing time. We envision that our assay can be developed for similar applications – assessing oral microbial profiles in dental clinics to assist dentists in the prevention and treatment of oral dysbiotic diseases – and could be utilized in a much more rapid manner with less labor required. For further ease of use, SHERLOCK has been previously employed on laminar flow strips and may be similarly applied for the detection of oral bacteria [17].

To accomplish the detection of oral bacteria using SHERLOCK, we solved multiple challenges specific to oral saliva samples such as computationally designing crRNAs that target oral bacteria, preparing saliva samples for SHERLOCK detection without a DNA isolation step, and testing the specificity of our crRNAs against a background of gDNA from human cells and hundreds of oral microbes. Although saliva contains numerous compounds that could interfere with SHERLOCK detection, such as endogenous RNases, off-target nucleic acid sequences, and salivary mucins, we did not observe any interference in our results using the SHERLOCK-EGTA+DTT methodology compared to SHERLOCK on isolated gDNA (Figure 3b and c). Previously, variations of this methodology have been combined with SHERLOCK or other Cas-based techniques to detect viruses and eukaryotic parasites in saliva [19,55,56], but they have not yet been applied to the detection of bacteria in saliva. Going forward, the addition of EGTA and DTT or similar adjuvant reagents to saliva samples may be critical to the future application of SHERLOCK and other nucleic acid-based detection systems to the oral field.

Limitations of our study included the testing of clinically healthy subjects without comparison to diseased patients. For this study, which was focused on establishing sensitive and specific detection, we reasoned that using healthy subject samples was sufficient. In addition, we observed that not all crRNA-primer sets displayed the same level of fluorescence when in the presence of equal amounts of target gDNA or live bacteria. These data suggest that in the case of gDNA detection, some crRNA-primer sets may display a higher efficiency than others. This could be due to RPA amplification, T7 transcription, crRNA detection, or some combination of the three steps. Further optimization of our crRNA-primer sets may prove fruitful in achieving higher fluorescent signals and faster detection times. Future studies will focus on testing additional crRNA-primer sets for each bacterial target. In addition, alternative isothermal amplification techniques to RPA, such as loop-mediated isothermal amplification [57] may display higher efficiencies and specificity.

Human oral diseases that have an etiology in microorganisms are generally accepted as polymicrobial in nature. Commensal microorganisms naturally present in the oral cavity, for instance, may become more abundant leading to dysbiosis and disease. Specific bacteria such as *P. gingivalis* and *S. mutans* are strongly associated with periodontitis and caries and are typically considered pathogenic [27,58]. Therefore, it is important to detect individual opportunistic pathogens and to profile the overall community. Within this scope, we have clearly illustrated in this study that SHERLOCK has the capability to detect specific oral bacteria and, with further development, may be capable of quantitating abundance (Figure 2f). Further, previous efforts to multiplex SHERLOCK for the detection of multiple organisms simultaneously have been successful [17,59]. The CARMEN system, for instance, is capable of simultaneously detecting 100+ human viruses [59]. Although not shown in our study, SHERLOCK detection has the potential to be expanded to other microorganisms in the oral cavity including viruses, fungi, and additional bacteria. Looking forward, SHERLOCK has the necessary capabilities (detection, quantitation, and multiplexing) to allow for the rapid profiling of an individual’s oral microbiome. Our current findings provide a foundation for the use of SHERLOCK and possibly other CRISPR-Cas based detection technologies in the oral research and clinical fields and are an important step forward toward individualized and precise oral care.

## Supplementary Material

Supplemental Material

## Data Availability

All nucleic acid sequences are provided in the manuscript or in Appx. Table A2. Raw data for Figures 1d-A4 and all code used in this project are available on Zenodo (DOI: 10.5281/zenodo.7708546). The raw data and code are also available at https://www.borlab.org/resources. Bacterial strains used in this paper will be provided upon request.
