# Supplementary material for: Rapid specific detection of oral bacteria using Cas13-based SHERLOCK
Source: J Oral Microbiol. 2023 May 11;15(1):2207336. doi: 10.1080/20002297.2023.2207336 (PMC10177689; doi:10.1080/20002297.2023.2207336)
Supplement: Supplemental Material [file ZJOM_A_2207336_SM5777.zip › Supplementary files/APPENDIX legends.docx]

**APPENDIX**

**Appx. Figure 1. a-f.** Fluorescence trace plot displays the specificity of the Pg (a), Kp (b), Sw (c), Sa (d), Ab (e), Aa (f) crRNA-primer set against other bacterial targets. A summary of each fluorescence signal at 35 min is shown in Figure 2b.

**Appx. Figure 2. a-f.** Fluorescence trace plot displays the specificity of the Pg (a), Kp (b), Sw (c), Sa (d), Ab (e), Aa (f) crRNA-primer set against background saliva gDNA. A summary is shown in Figure 2e.

**Appx. Figure 3. a.** SHERLOCK detection at 35 minutes of decreasing 10-fold concentrations of Sm gDNA alone. **b.** SHERLOCK detection at 35 minutes of decreasing 10-fold concentrations of Sm gDNA alone combined with 10 ng of saliva gDNA. A summary is shown in Figure 2f.

**Appx. Figure 4. a-b.** Fluorescent trace produced by SHERLOCK with Sm (a) or Pg (b) gDNA and SHERLOCK-EGTA+DTT spiked with an equivalent concentration of live Sm (a) or Pg (b) cells. A summary of these plots is shown in Figures 3b and 3c.

**Appx. Figure 5. a-g.** Specificity and fluorescent signal of crRNA-primer sets when used with SHERLOCK-EGTA+DTT and unprocessed saliva samples spiked at a concentration of 300 Pg (a), Kp (b), Sw (c), Sa, (d), Ab (e), Aa (f), Sm (g) bacterial cells per reaction. A summary of these plots is shown in Figure 3d.
