# Supplementary material for: Rapid specific detection of oral bacteria using Cas13-based SHERLOCK
Source: J Oral Microbiol. 2023 May 11;15(1):2207336. doi: 10.1080/20002297.2023.2207336 (PMC10177689; doi:10.1080/20002297.2023.2207336)
Supplement: Supplemental Material [file ZJOM_A_2207336_SM5777.zip › Supplementary files/Appendix Tables.docx]

**Appendix Tables**

Appendix Table 1. List of reagents for one-pot SHERLOCK and their respective volumes per reaction.

| **Components** | **Volume / reaction (μl)** |
| --- | --- |
| Forward primer | 1 |
| Reverse primer | 1 |
| Rehydration buffer | 11.8 |
| Cas13 enzyme (0.5 mg/mL) | 0.32 |
| crRNA (1 μM) | 0.56 |
| Reporter (2 μM) | 1.56 |
| RNase Inhibitor (40 U/μl) | 0.63 |
| rNTP mix (25 mM) | 1 |
| T7 RNA Polymerase (50 U/μl) | 0.5 |
| MgCl2 (1 M) | 0.23 |
| MgOAc (280 mM) | 1 |
| H2O | 1 |
| RPA Pellets | 0.33 |
| Total Volume | 20.6 |

Appendix Table 2. Sequences of designed RPA primers and crRNA for targeted bacteria.

| **Species** | **Forward Primer (5'-3')** | **Reverse Primer (5'-3')** | **crRNA (5'-3') (RNA)** | **Target Gene UniRef90 Accession** | **Target Gene Sequence** |
| --- | --- | --- | --- | --- | --- |
| Sm | AATTCTAATACGACTCACTATAGGGTCCAGTAGATGGGCATTTATCTTTATTATCACGA | GGCAAGTCCAAAAATAAAATAAGGATTGAA | GGGGAUUUAGACUACCCCAAAAACGAAGGGGACUAAAACAUAACACAAUGAAUAAAAAAUGACUUGA | Q93ES7 | ATGTCTATCTTTAAAAATAAATTATTTGTCATTTTATTTGCCTTTCTTATGTTACTTGTAGATGGGCATTTATCTTTATTATCACGAATCTTATTTCAAAATCAGTTTATTGTGTCAAGTCATTTTTTATTCATTGTGTTATTGTTTTATACTCTTGTATTCAATCCTTATTTTATTTTTGGACTTGCCTGTGTTTTAGGAATTATTTATGATTTTTATTATTTAGGGGTATATAATTTGGGAATTGCAACTATGTTGTATCCTTTAACAATTGTTATTATGTTTAAATTATGGAAACATATTCCAAATGGTCCTGTTCAACGCTTTCTAGTTTTTTTTATCCTAATCTTTTTTCTTGATTTTGCTAGTATTGGTATGGCCTATTTATATCAATTGACAGCTTATCCTTTAAATGATTTTATCACTTATAATTTAGCACCTTCCTTAATTTTTAATATACTGGCTTTTCTCTTCTTTCAAAAACTTTTAGAAAGGATTTACCTATGA |
| Sw | AATTCTAATACGACTCACTATAGGGTCCATTCCCTTATTCTTTCTTATTTCCTCTGTTT | GAAGAGGAATAAGGATGAAGATGAGATGAG | GGGGAUUUAGACUACCCCAAAAACGAAGGGGACUAAAACAGAAAAAAGAAGAAUAAGAAUAAUAUGA | J0LL06 | ATGATAGTTAAGGGGCGGGAAATAATCCCGCCCCTTAACTATTTCCCTTATTCTTTCTTATTTCCTCTGTTTTCATATTATTCTTATTCTTCTTTTTTCTTTTTACCCTTTCGCACTCGCCCTCTTCACCTGCGTTCTCCTTCTTCCTTCCCTCATCTCATCTTCATCCTTATTCCTCTTCATCCTCGCTCCGGTCGTCATCCCGCTTCACCTTCCAATCTCACTCTGCCCTTCCTTACACCCTGCCTTTTCTTACTCTCCAATCTCATCTCACCTCCTTATTTTTCGATCTCACCCCGTTCTTCCTTACTCTCCGATTTCATCTCACTTCTTTACTTTCCGACCTTTTATCCACATACATATGTAGCCCCACTTCCGACCTCACTCCATCTCTCCCTACCTTCTGACCTCACTCCATACCCTCCTTACTTTCCGATCGTACTCCACCATCTCCTTACTCTCCAATCTCACCCCATCCCCTCACCCAACTCGAAGATACGGATCCTCCTCCGACGGCCTCCTCCCACATCCGTACAGCTGAAACTATAA |
| Aa | AATTCTAATACGACTCACTATAGGGTCCACCCTTTGTTAAAAACGATAGCAGAAAAATA | TGCAATATGAATAATAGAGGTATCAGGAGA | GGGGAUUUAGACUACCCCAAAAACGAAGGGGACUAAAACAUAACGAGGCUAGUAUAAUGAAGUAAUG | A0A142G015 | ATGAAAGCATTACTCCGAAAAATCCGTTTAGCCTTAGGCAAAATGTTGCTAGACAAAAATGTTCAGGGGCAGGCTTTGCCGGCAAATCCGAAAATTATTGTGTTACAACAAGACGGAAAAATCGGAGATTATATTGTCAGTTCATTTATTTTCAGAGAATTAAAACGACATAACCCTAAAATGCAGGTTGATGTTGTATGTTCACCTAAAAATGTCAATTTATTTGAACAAAATCCATCTATTGATCATTGTTTTATTTTAAATAGAAAAGAACATTGCGCTTACAGCAGAATGGGAAAACAACTTTCACATGAACATTATGATGTATTAATTAATTTACCGGTATTATTACGGAATCGTGATTTGTGGCTAACCCGATTAATTCATGCAAAAAATAATATCGGCTATAAAAAACAAAACTACAAACTATTTAATTTGAATGTTACTCAAGATCAATTACATTTTTCCAAAGTTTATGCAGAAGCCATTAAATTATGTGGTGTAAAAGATATTAATCTTGAATATGACATTCCAAATCACAGTGATAAAAAAGAAGATATTGCAAATTTCATACAAAAAAATCATCTTGTCGATTGCATCGCTATAAACTTTTTTGGTGCAGCCGGCACCAGAAAATTTACGGAACAAAACATTTATCGGTTTATGGAAAAATTTAAAGCCGAAAATAAAAAAGCGCTGTTACTAACTTACCCCGAAGTAACCCCTTTGTTAAAAACGATAGCAGAAAAATATACCAATGCTTTTATCTATGAAAATACTGAAAATATCTTTGATACGATAACATTACTTCATTATACTAGCCTCGTTATTTCTCCTGATACCTCTATTATTCATATTGCAGCAGGATTAAATAAAAAAATTATTGGATTTTATAAGCTCGCTGATAAAGAAAACTTTACGCATTGGAATCCAAACTGTAAAAATAAAACATATATCCTTAATTTTATTGAAAATGTCAATGAAATCTCTCCCGATGAAATTAAATCAGAATGGTTAAAATAA |
| Pg | AATTCTAATACGACTCACTATAGGGTCCAGATTTTCTTATCGGATTTCACAAATGACTG | AATCACTTTTTCACTCGTATAGGTCAGTTG | GGGGAUUUAGACUACCCCAAAAACGAAGGGGACUAAAACAAGUGGACAAAGGAGAUAAGAUACAGCA | B2RJX1 | GTGGCAAAATCCGATAGAACGTTTTTAATGCCGGTTACGATATTGATCGCCCTCGTGTGTAGGATGCTGTCCTACGCATGGGGGCTTTCTGCTGATATTTCTCTTCAAGAGTGTAGTCTTCCCCTCTTCGGCACGATCCCTTTCTTATGGCAAACCGTTATCTCGTTTGCCATTGGCTTGGTTGCATCTTTTATTGCGGTTCGCTTCAGTGCTTTCTATCTTCTGCTTCATGAAGGTGGATTCCGGCCATTTGCTTTCTTGATGATTCTTTTGCTGAACACCCATCAGGTTTTCTTCCCCATGCAGCCATACTCGCTGTCGATACTGCTGCTGATAGTACTCTTTTTCTGCCTTTTTGGCACTTATGGCCGTAATAATATCCCGCCCAAGATGCTTAACGTAGGCTTTTGTGTCGGGCTTTCGGCTGTGTTGTGGTCTCCTTCGCTGCTTCTTGCACCGTTTGTGCTCATACAGTTTTATCTGATGAAGAGTTTGTCTTTCAAAAACTTGATTGCATTTTTCTTCGGACTGTTGCTTCCTCTGTGGTGCTGTCTTCCTTTATTGGTATTGGCCGGACAGGAGCAGTTTGTTATCGACAATATGACCCTTTTGACTCGGTGGGGATTTTCTTATCGGATTTCACAAATGACTGCGTGGAAGGGGCTGTATCCGGCTCTACTGTCAGTGCTGTATCTTATCTCCTTTGTCCACTTGCAACTGACCTATACGAGTGAAAAAGTGATTGCACGCATTTACTTCTTCAGCCTTTTGTGTGCCGGTTGTTATATGCTGCTACTCTCTGCCGTGATGCCTGCTGCTTCCGAAGGATTCGTATATCTGGCTACCTTCCCTGTGTCGGTACATGCAGCTCGTTTCTTGAATTCTCTGAATCGTCGTGTTGCCAATATCTTGATTCCTTTTACGTTTTTGGTTTTCTTGTCCTCTTTTTTGCTGTCTTCTTTTTTCCTTTAG |
| Ab | AATTCTAATACGACTCACTATAGGGTCCATACAATAATGCTAGATTTATCGCACTAGTG | GCAGTACAAATGAAAATTGAACCAGATTTA | GGGGAUUUAGACUACCCCAAAAACGAAGGGGACUAAAACAAAUUAUUGAAAAUAUAGCAAACAGACC | A0A0E1FMW9 | ATGGCTAAACGCTACTTACCCTTCTACAATAATGCTAGATTTATCGCACTAGTGTTAGTCGGTCTGTTTGCTATATTTTCAATAATTTTTAAATATTTAGAGTTAAATATTACAATAAATCTGGTTCAATTTTCATTTGTACTGCTTCTCCCTTTAAGTCAAATTTATTTGGCTTATAAAGGTATGCTCGATGCATTAAAGCTTGATGGTTTAAATCAGTCAGAACGAGATAGGTTGACTTCAACTGTGGACATAAGAAGTAAGTCATCTTTATATGTGGCTATGCTTTTTATTATTCTTGTTTTTAGTATGTATATACTTAATTTATTAGGCTTACTTTCAGCTAAGCATCTTTTAGCTCTAATACTTTCTGTTGGACTCACCTCAATTTTTAGCTTCTTCTTAGCTTGGTCTGACTTAAGAGAAATCTCTTTGCTTGAAAAAACATTAAAAGATCGCAAAGAATCAAGAGAGGCAAAAGCAAAAGTATTGAGCAATAAGTAA |
| Kp | AATTCTAATACGACTCACTATAGGGTCCAGATACGATGATCTTCAGGCGTTATACAATG | GATTTTATAAATTGATGCCATCTGCTGATT | GGGGAUUUAGACUACCCCAAAAACGAAGGGGACUAAAACAUUUUUUAAAUAAACACUGCCAUCACUG | A0A1D3KD86 | ATGAAGACGTTGACTTTCCTGCTTTCAACGGTCATTGAGCTTTACACGATGGTCGTGTTGTTACGCGTCTGGATGCAGTGGGCGCGCTGCGACTTTTACAATCCGTTTTCGCAGTTTGTGGTGAAAGCCACGCAGCCGATTGTCGGACCGCTGCGCCGGATTATCCCGGCAATGGGGCCGATTGACAGCGCCTCGCTGCTGGTGGCATTTATTCTCTGCGTCATCAAAGCGATCGTGCTGTTTATGGTGATCACCTTCCAGCCGATCATCTGGATCTCCGCTCTGCTTATTCTGCTGAAAACCATCGGCTCGTTGATCTTCTGGGTACTGCTATTGATGGCGATCATGAGCTGGGTCAGCCAGGGCCGTAGCCCGGTGGAGTATGTGCTGATGCAGCTGGCGGACCCGCTGCTGCGCCCTATCCGCAACCTGCTGCCGTCGATGGGCGGTATCGATTTCTCGCCGATGGTGCTGGTACTGCTGTTGTACGTAATCAATATGGGCATCGCCGAAGTGCTGCAGGCCACCGGTAATGTACTGCTGCCGGGGCTGTGGATGGCGCTATGA |
| Sa | AATTCTAATACGACTCACTATAGGGTCCAGAAACCTTTGCTATAATACCTATCGTCTTT | AAGCGACAATCTAACATTAAAGAAGTGATA | GGGGAUUUAGACUACCCCAAAAACGAAGGGGACUAAAACAAAAAUAAGCAGAAAAACAUUAUGUAGUU | A0A386C2Q6 | TTGGTAGTATTGTTATCTTATAGTTGCAGTTGTATCTTTAGTTTTCTATATCAATTTATATCTATAGAAGAAACGTCATTTGATTATTTACATAGAAGATCGAAATGTGATTATTGTAATTCATCACTCAAATGGTATGAATTAATGCCGATTATTAGTTTTTTATTATTAAAAGGGCGATGTCGAAACTGTCGAAAGCGTATTTCCCTAACACATTTCTTAGGGGAAACCTTTGCTATAATACCTATCGTCTTTATTAAGTATGATTTCACATACGTAAATGCTACGCTATTTATAACTACATATGTTTTTCTGCTTATTTTTACTATGACCGATATCACTTCTTTAATGTTAGATTGTCGCTTAATTATAATTTATTGTATCGTTTCTCTCTCGTTAAGTATGATTTATCCAGTAGCTTTTATCATTATTAGTATGACCACGCATATATTCTACTTTTTATTTCGGGCATATATTGGTTATGGTGACGTTTTACTAATATCTGCACTTTCTTTGTTTTTCCCTCTCCAATTCACTATTTATGTCATTTTATTTACATTTGTCATTGCTGGTTTAGTTGCTTTAATTACCATGATATTTAAGCCGATTAAACTATTACCCCTTGTTCCATTTATATTTATTTCATTTTTTATCAATTCACTTTTTTATAATGATATCCATCAATTTTTAGGAGGCGTATATTTTTGA |
