# Supplementary figures and images for: Rapid specific detection of oral bacteria using Cas13-based SHERLOCK
Source: J Oral Microbiol. 2023 May 11;15(1):2207336. doi: 10.1080/20002297.2023.2207336 (PMC10177689; doi:10.1080/20002297.2023.2207336)

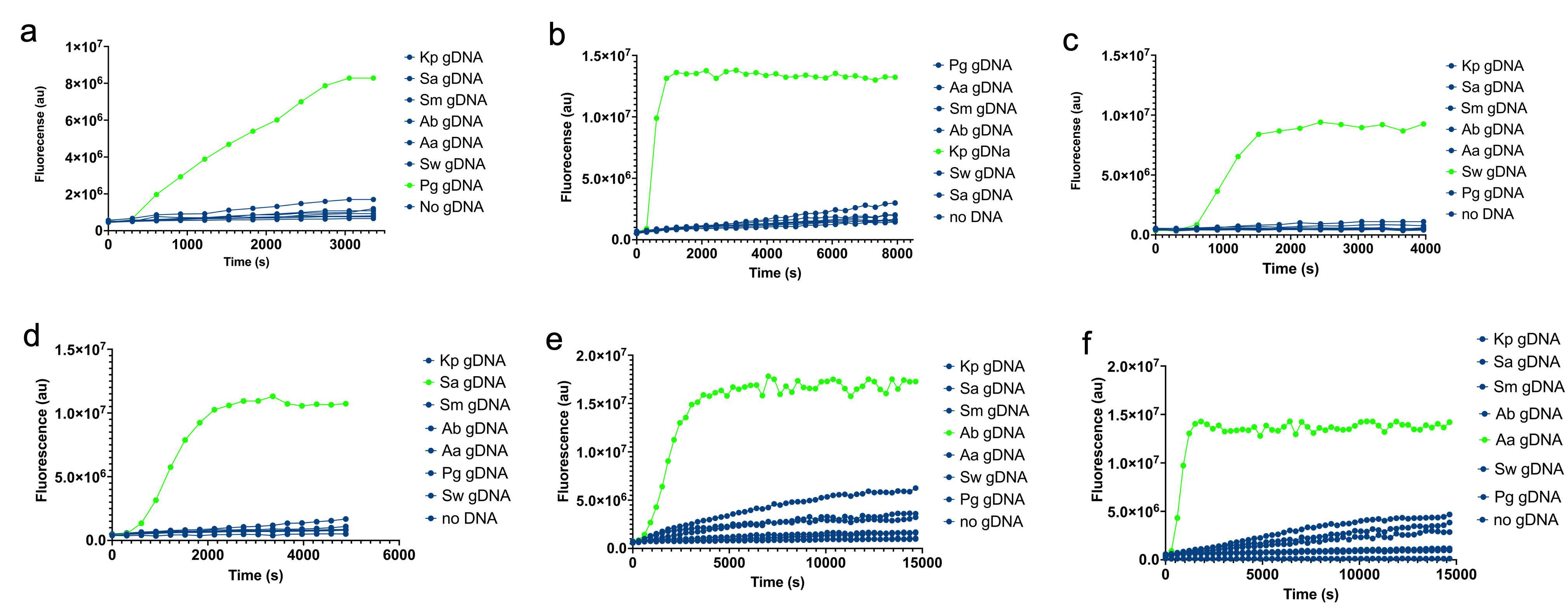

Supplement: Supplemental Material [file ZJOM_A_2207336_SM5777.zip › Supplementary files/AppFig1_High_Res.jpg]

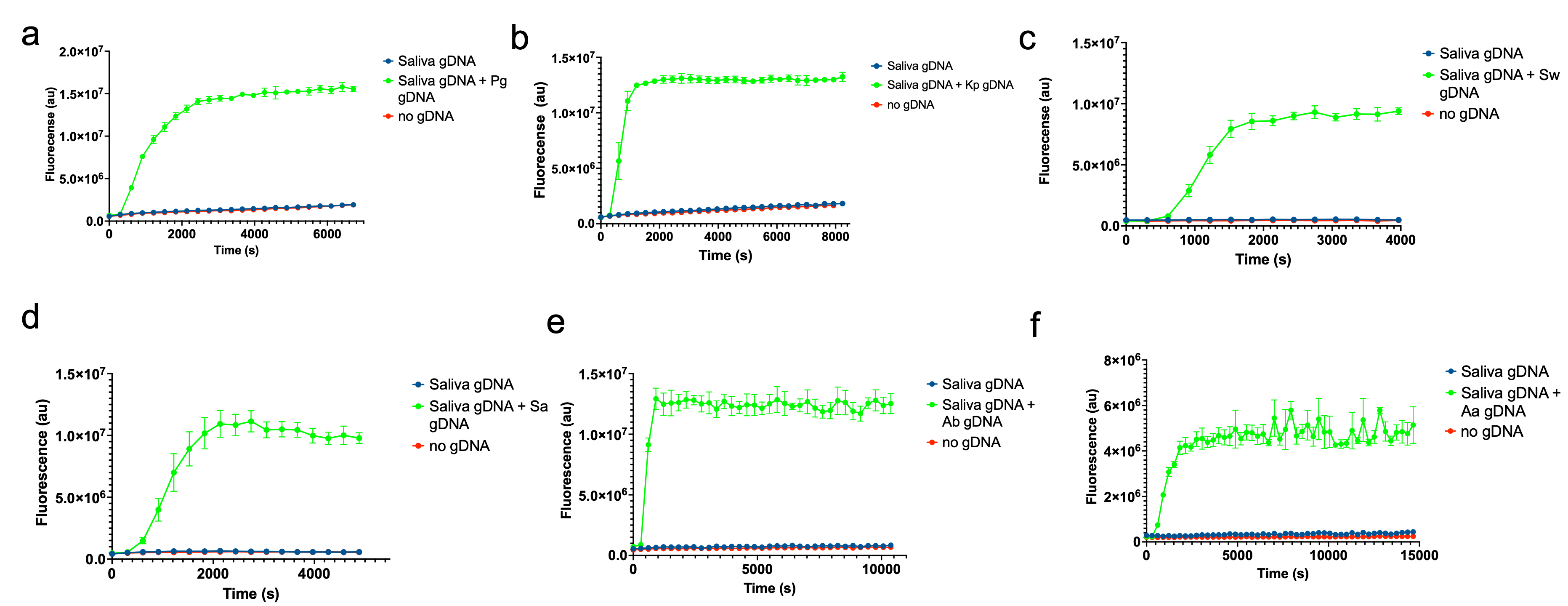

Supplement: Supplemental Material [file ZJOM_A_2207336_SM5777.zip › Supplementary files/AppFig2_High_Res.jpg]

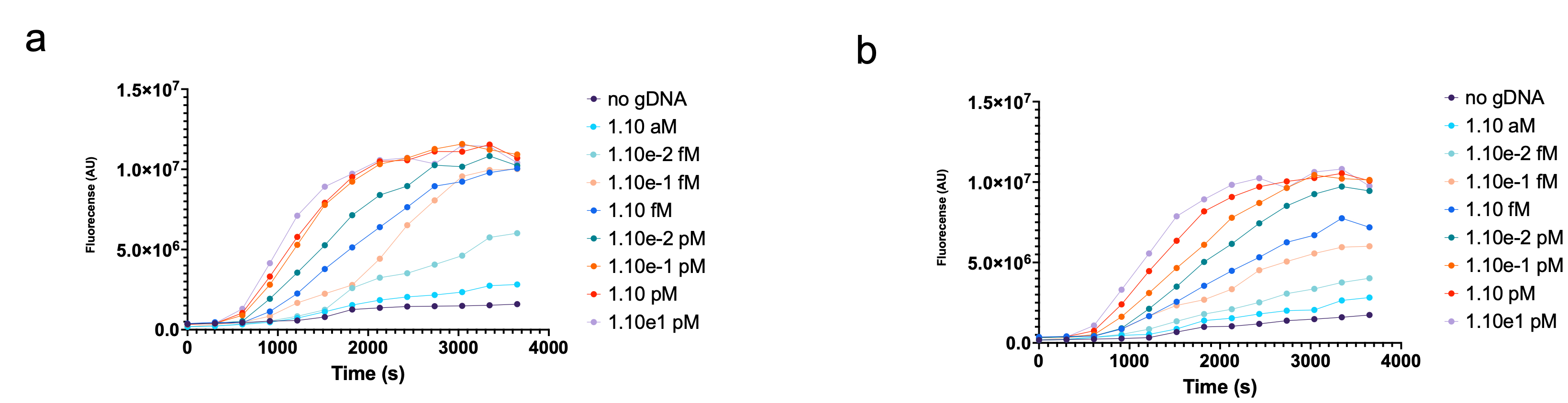

Supplement: Supplemental Material [file ZJOM_A_2207336_SM5777.zip › Supplementary files/AppFig3_High_Res.jpg]

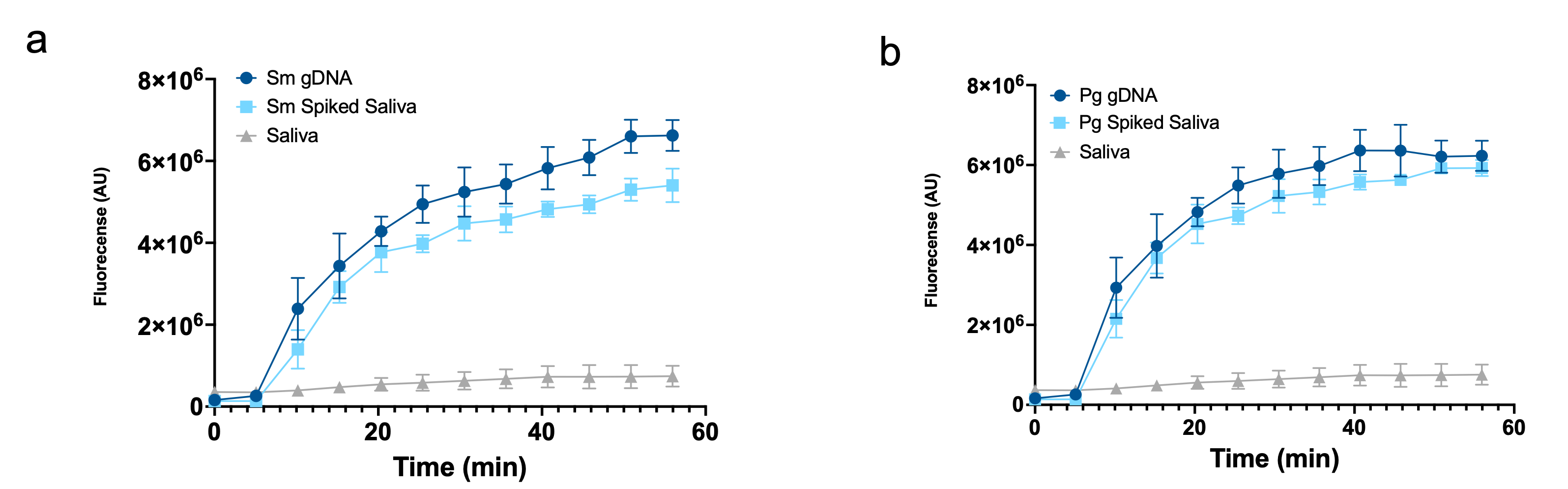

Supplement: Supplemental Material [file ZJOM_A_2207336_SM5777.zip › Supplementary files/AppFig4_High_Res.jpg]

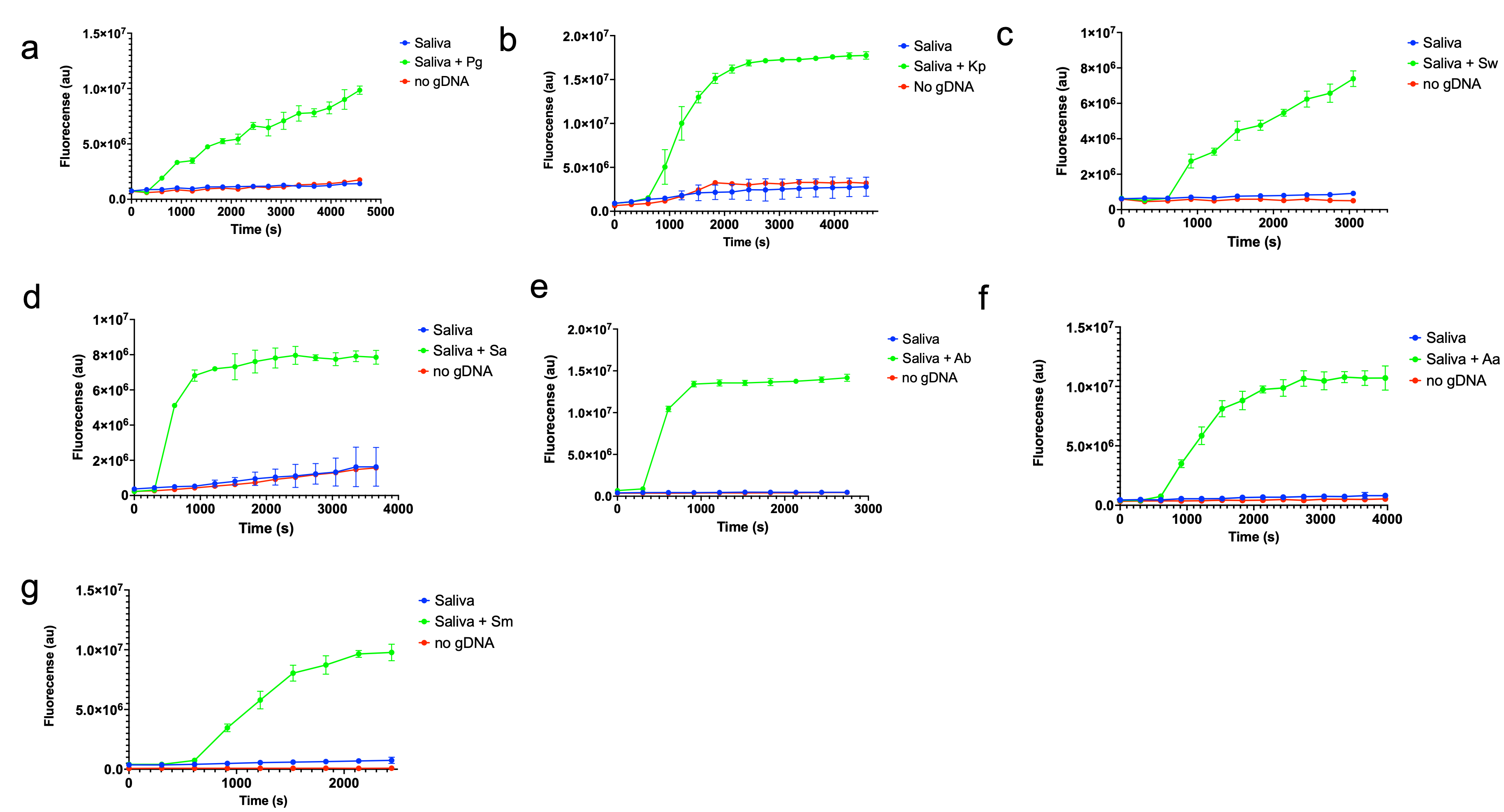

Supplement: Supplemental Material [file ZJOM_A_2207336_SM5777.zip › Supplementary files/AppFig5_HIgh_Res.jpg]
